# Alternative Splicing in the Hippo Pathway—Implications for Disease and Potential Therapeutic Targets

**DOI:** 10.3390/genes9030161

**Published:** 2018-03-13

**Authors:** Sean Porazinski, Michael Ladomery

**Affiliations:** Faculty of Health and Applied Sciences, University of the West of England, Frenchay Campus, Bristol BS16 1QY, UK

**Keywords:** Hippo pathway, alternative splicing, yes-associated protein, splice-switching oligonucleotides, gene therapy

## Abstract

Alternative splicing is a well-studied gene regulatory mechanism that produces biological diversity by allowing the production of multiple protein isoforms from a single gene. An involvement of alternative splicing in the key biological signalling Hippo pathway is emerging and offers new therapeutic avenues. This review discusses examples of alternative splicing in the Hippo pathway, how deregulation of these processes may contribute to disease and whether these processes offer new potential therapeutic targets.

## 1. Introduction

The Hippo pathway is a newly established serine-threonine kinase cell signalling pathway implicated in several key developmental and homeostatic processes. Deregulation of the Hippo pathway is thus associated with a suite of disease states including a variety of cancers such as breast, pancreatic, prostate, liver, lung, bladder, gliomas, melanomas, sarcomas, colorectal, ovarian, gastric and head and neck cancers (reviewed in [1]). As such, this emerging pathway and its components have garnered considerable interest in recent years as a potential therapeutic target. Many of the core proteins constituting the pathway are now well established and some of its earliest identified biological roles include the control of cell proliferation and growth; however, the full repertoire of key components, interactions and functions of the Hippo pathway are still being discovered. In particular, a role for alternative splicing as a means for regulating the Hippo pathway is emerging and remains to be fully understood. This review will briefly introduce the mechanism of alternative splicing and summarises what is known about the functioning of the Hippo pathway, before moving on to examine emerging evidence for a role for alternative splicing in the functioning and regulation of the Hippo pathway. We will then explore the notion that aberrant alternative splicing of components involved in the Hippo pathway could contribute to disease states and ask whether or not alternative splicing in the Hippo pathway provides novel therapeutic targets.

## 2. Alternative Splicing and Disease

**Figure 1:**
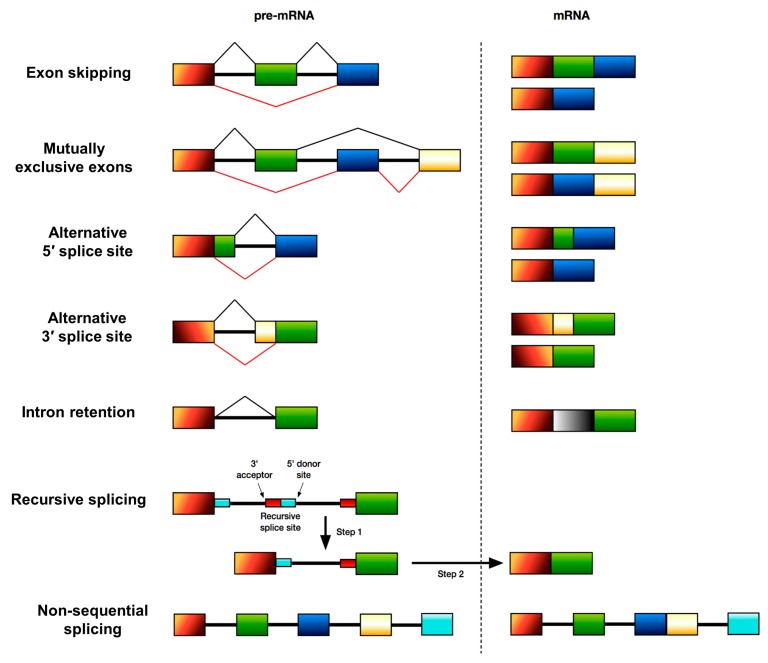
Principal types of alternative splicing of human genes. Pre-mRNA is shown on the left, and the mature product of the splicing process is shown on the right. Black and red lines indicate differential splicing outcomes.

Alternative splicing (AS) was discovered over 35 years ago and is an important regulator of multicellular eukaryotic gene function that allows for the production of multiple mature mRNAs from a pre-mRNA. This means that a single gene can encode multiple protein isoforms with often opposing functions. It is estimated that 95% of human genes are alternatively spliced [2], highlighting the importance of AS in the regulation of gene expression. AS is a co-transcriptional process involving the differential inclusion or exclusion of particular exons of a gene in the final mature mRNA molecule. AS patterns differ in specific tissues [3]; for example, the central nervous system in particular relies heavily on AS to produce proteins required for its proper functioning. There are several principal types of AS of human genes (summarised in Figure 1); these include skipping or retention of cassette exons, the use of mutually exclusive exons, the use of an alternative 5′ donor splice site, the use of an alternative 3′ acceptor splice site, intron retention, recursive splicing [4] and non-sequential splicing [5].

AS processes are highly conserved between taxa but the extent of AS differs across organisms and is more prominent in multicellular organisms [6]. Whereas in humans 95% of genes are alternatively spliced [2], recent reports suggest that there are only a few examples of functional AS in the budding yeast *Saccharomyces cerevisiae* [7,8] and that only two to three percent of genes in the fission yeast *Schizosaccharomyces pombe* are alternatively spliced [9,10]. In contrast, in *Drosophila melanogaster* 60% of genes are alternatively spliced [11]. AS is involved in regulating a variety of biological processes including cell division and apoptosis [12], differentiation during development and in adult tissues [3] as well as how cells respond to external stimuli (e.g., [13]). It is therefore not surprising that AS has been implicated in regulating key signalling pathways such as Ras-MAPK and PI3K-mTOR signalling [14]. Aberrant AS is a major contributor to several neurological diseases including Duchenne muscular dystrophy (DMD) [15], spinal muscular atrophy (SMA) [16], diabetes [17] and is implicated in the development and progression of many types of cancer [18,19]. As such, there has been a lot of interest in using splice isoforms as disease biomarkers and in developing novel therapeutic strategies aimed at oncogenic splice isoforms [20,21].

## 3. Introduction to the Hippo Pathway

**Figure 2:**
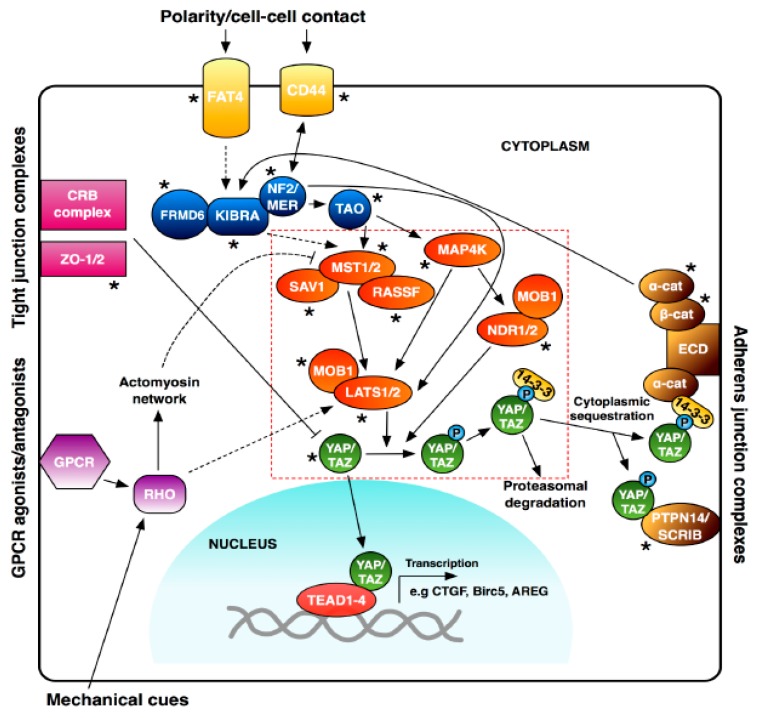
An overview of the mammalian Hippo pathway. Hippo pathway members shown inside the red dashed box are considered the core components of the Hippo pathway. Members with an asterisk are those known to have alternative splicing variants. Dashed arrows indicate unknown mechanisms.

The Hippo pathway is a recently discovered cell signalling pathway implicated in key biological processes. Despite only just over 20 years of research, the Hippo pathway has become established as the main essential regulator of organ size, but new functions for this important pathway and its components are still being elucidated. Whilst many of the core genes involved in the Hippo pathway were discovered in *Drosophila* due to the overgrowth phenotypes generated by their mutation [22,23,24,25,26], the pathway is highly conserved in mammals via orthologous genes. In mammals the pathway consists of a core cassette of upstream serine-threonine kinases and adaptor proteins that serve to negatively regulate the downstream effectors of the pathway (see Figure 2 for an overview), yes-associated protein (YAP) and its paralogue, transcriptional co-activator with PDZ-binding motif (TAZ). YAP and TAZ act as transcriptional co-activators to elicit expression of target genes regulated by the Hippo pathway [27]. The activation of upstream Hippo components leads to the subsequent phosphorylation of YAP/TAZ and their cytoplasmic sequestration blocking their activation of target genes in the nucleus [27]. YAP/TAZ nuclear activity is achieved through binding with several transcription factors, most prominently TEA Domain transcription factors (TEADs), but other examples include RUNX transcription factors [28], SMAD proteins [29,30], FoxO [31] and p73 [32]. 

As such, the Hippo pathway interacts with a host of other signalling networks, including the WNT pathway [33,34], Ras signalling [35], metabolic pathways [36,37,38,39] and pluripotency/stemness pathways [40]. Due to this cross-talk, there are many regulatory inputs into the Hippo pathway including those that regulate cell polarity and adhesion [30,41], cell-cell contact [41,42], mechanical cues such as extracellular matrix (ECM) stiffness and cell geometry [43,44] soluble factors such as hormones and growth factors through G-protein-coupled receptors (GPCRs) [45,46,47], chemical stresses [48] as well as metabolic changes [49]. Consequently, to date the Hippo pathway has been implicated in a diverse array of biological processes, the coordination of which is essential in multicellular organisms for regulation of organ size. These processes include cell proliferation [50], apoptosis [51,52,53], epithelial-to-mesenchymal-transition (EMT) [54] and cell migration [55,56], cellular plasticity [57], stem cell biology [58], coordinated morphogenesis of tissues and development of three-dimensional body shape [59] and regeneration [60]. In the liver, over-activation of YAP for four to five weeks was shown to increase organ size four to fivefold [61,62], and over-expression in the pancreas also caused an enlargement [61]. Furthermore, YAP over-activation has been shown to cause epidermis thickening and dysplasia of the intestinal epithelium [61]. The requirement of YAP in vivo is underscored by the fact that homozygous deletion of YAP causes developmental arrest in mice by stage E8.5 accompanied by defects in yolk sac vasculogenesis, a notably shortened body axis and a convoluted anterior neuroepithelium [63]. 

## 4. Examples of Alternative Splicing in the Hippo Pathway

Recent work has shed light on the evolution of the Hippo pathway, suggesting that the manner in which Hippo pathway genes have developed allows for species-specific control of growth [64]. For example, most of the Hippo pathway genes can be found in even the simplest metazoans despite their basic body plans but Hippo genes exhibit more exons in advanced organisms where tissues are multicellular and more complex in nature [64]. A study by Zhu et al. [64] found that although the exon number of Hippo genes varied between species, functional domains were often highly conserved, suggesting that Hippo pathway genes may be able to produce multiple protein isoforms by AS to allow growth control across a variety of different tissues. Indeed, it appears that the functional domains of Hippo pathway components have co-evolved with their interacting partners’ functional domains, undergoing purifying selections (i.e., selective removal of domains that do not allow the protein to achieve its function), highlighting the evolutionary importance of the pathway [64]. This study concluded that AS might be the most important feature of Hippo pathway genes to produce multiple proteins for tissue-specific growth control [64]. 

Whilst there is still much to learn regarding a role for AS in the Hippo pathway, there are several lines of evidence suggesting AS is involved in regulating Hippo signalling. It remains to be seen whether or not AS plays a more prominent role in regulating Hippo signalling versus other key signalling pathways, but it is conceivable that AS could provide a more stable type of regulatory mechanism versus the fast regulation afforded by the traditionally well-studied phosphorylation cascade of the Hippo pathway [65]. The majority of the core components of the Hippo pathway are known to have alternatively spliced isoforms (Table 1). The mammalian Ste20-like kinase-1 (*MST1*), a serine/threonine kinase with a SARAH domain has 21 splice variants, with only two being protein coding. The C-terminal truncated version of MST1 consists only of the kinase domain, whereas the full-length MST1 isoform consists of the C-terminal regulatory region as well as the homodimerisation SARAH domain [66]. SAV1 (a protein containing two WW domains, a SARAH domain, and a coiled-coil region [67]) has six splice variants, with five of them producing proteins and *MAP4K* (belonging to the mammalian Ste20-like family of serine/threonine kinases) has 15 transcripts with five of them producing proteins and one undergoing nonsense-mediated decay (NMD). The kinase co-activator MOB1 [68], also has six splice variants with just one transcript producing a 216-amino acid protein. Finally, large tumour suppressor kinase 1 (LATS1), a serine/threonine kinase has seven transcripts with four being protein encoding and two resulting in NMD whilst large tumour suppressor kinase 2 (LATS2), also a serine/threonine kinase has two splice variants, one being protein coding. 

*YAP* is comprised of nine exons and can undergo AS, and to date there are at least eight described splice isoforms of the *YAP* gene expressed in human tissues [69]. These different YAP isoforms can have different functions in different tissues as each version varies in transcriptional potency [70]. Furthermore, the different YAP isoforms can interact with different binding partners in different contexts. For example, exon 4 skipping within *YAP* results in a protein with only one WW protein binding domain as opposed to the more frequent product that contains two WW domains [71,72]. WW domains are commonly occurring protein-protein interaction modules that recognise proline-rich motifs [73] and the WW domains of YAP are crucial for the interaction of YAP with LATS protein kinases [74]. There are several functional consequences of YAP having only one WW domain, one being that YAP can no longer bind angiomotin, a member of the tight junction complex, which is normally involved in sequestering YAP in the cytoplasm [75]. Additionally, YAP can also no longer bind p73 without the second WW domain, meaning it can no longer exert its pro-apoptotic functions under conditions of physiological stress [76]. *YAP* exon 5 has an alternative splice donor site producing a longer transcript (exon 5B), and together with cassette exon 6, can vary the transcriptional activation domain (TAD) of YAP between β, γ, and δ isoforms depending on exon 5 and 6 inclusion/exclusion [69]. This in turn affects the transcriptional activity of YAP [70]. Novel neuron-specific YAP isoforms were recently reported by the Okazawa group [77] and found to be present during a specific type of slow and progressive neuronal death known as transcriptional repression-induced atypical death (TRIAD), associated with Huntington’s disease (HD) [77]. These three YAP isoforms (ins13, ins25, and ins61), referred to as YAP∆Cs, are C-terminal truncated isoforms of YAP and contain additional mini-exon sequences between exon 5 and exon 6 [77]. During TRIAD these YAP∆Cs functioned in a dominant-negative manner to suppress neuronal death and were found to be co-localised with activated p73 in striatal neurons of HD patients and mouse models of HD [77]. YAP∆C isoforms were also found to attenuate neuronal death in *D. melanogaster* huntingtin mutants [77]. YAP∆C-ins61 had the strongest anti-TRIAD activity and the therapeutic use of YAP∆C-ins61 in a mouse model of spinocerebellar ataxia type 1 (SCA1) found that expression of YAP∆C-ins61 in development, as opposed to during adulthood was required to ameliorate the pathology and symptoms of SCA1 (ataxin-1 knock-in mutant) mice [78]. Specifically, Fujita et al. found that YAP∆C-ins61 functions as a co-activator of the nuclear receptor retinoid-related orphan receptor alpha (RORα), forming transcriptional complexes with RORα on *cis*-elements of target genes to invoke gene expression necessary for cerebellar development [78]. Since the three YAP∆C isoforms all exhibit a reading frame shift, resulting in truncation of the C-terminal TAD, it will be interesting to see whether these isoforms are also present in other tissues, as loss of this C-terminal region would compromise YAP function in several ways. For example, loss of the YAP PDZ-binding motif associated with the C-terminus would impair the nuclear shuttling of YAP [79]. 

Very recently it was reported that YAP nuclear activity is under the control of a splicing switch. The TEAD4 transcription factor—the major binding partner for YAP in the nucleus—has an alternatively spliced isoform that produces a truncated version lacking the N-terminal DNA-binding domain [65]. This truncated TEAD4 isoform (TEAD4-S) is found in both the cytoplasm and nucleus where it can still bind YAP and acts in a dominant negative manner over full length TEAD4 to repress YAP activity in both locations. Qi et al. found that production of TEAD4-S is governed by the tumour suppressor and splicing factor RNA-binding protein 4 (RBM4) by direct binding of RBM4 to TEAD4 pre-mRNA causing skipping of exon 3 [65]. TEAD4-S levels in lung cancer cell lines and patient samples are reduced, suggesting that TEAD4-S may attenuate YAP signalling to suppress cancer cell proliferation. In support of this they found that by re-expressing TEAD4-S in two lung cancer cell lines in vitro they could reduce the proliferation of the cancer cells and reduce expression of the EMT markers *N*-cadherin and vimentin. The anti-tumour effects of TEAD4-S were also seen in vivo using xenografts of cancer cell lines where the truncated isoform slowed tumour growth [65]. Furthermore, in patient samples from lung and colon cancer that were examined for levels of TEAD4-S, it was found that those with higher TEAD4-S levels had improved overall survival [65]. 

It is also becoming apparent that downstream target genes of the Hippo pathway can be regulated by alternatively spliced isoforms of proteins that interact with the Hippo pathway [80]. Receptor tyrosine-protein kinase erbB-4 (ERBB4) has previously been shown to interact with YAP to induce expression of the Hippo pathway target gene *CTGF* [81]. However, Wali et al. determined that certain ERBB4 splice isoforms (CYT-1 or CYT-2) could affect expression of *CTGF*, as well as the additional Hippo target genes *CYR61* and *SPARC* [80]. Specifically, microarrays showed that only the full-length CYT-2 ERBB4 isoform regulated *CTGF* and *CYR61* expression in basal MCF10A cells (normal human mammary epithelial cells), whereas both CYT-1 and CYT-2 soluble intracellular domain (ICD) isoforms upregulated *CTGF* and *CYR61*. Both the CYT-1 and CYT-2 ICD forms of ERBB4 also upregulated *SPARC* expression, which was associated with increased cell proliferation and invasion in the case of the ICD CYT-2 isoform. Interestingly, the study found that in the luminal breast cancer T47D cell line, *CTGF* and *CYR61* were also upregulated by ERBB4 isoforms whereas *SPARC* was downregulated, suggesting a cell-type specific role for ERBB4 splice isoforms in regulating Hippo pathway target genes. In all cases, YAP was predicted to be the upstream transcriptional activator regulating the changes in *CTGF*, *CYR61* and *SPARC* [80]. These data provide interesting evidence of a link between growth factor receptors and the Hippo pathway and suggest that AS mechanisms extrinsic to the core Hippo suite of genes can also regulate pathway activity, adding another level of complexity and regulation to the Hippo pathway.

Recent work suggests that the downstream Hippo pathway components YAP and TEAD can regulate which splice isoforms of an upstream Hippo component, *KIBRA*, are expressed in certain tissues, potentially allowing tissue-specific tailoring of Hippo pathway function [82]. In this study, mapping analysis identified four novel and differentially used transcription start sites (TSS) within the *KIBRA* gene. These TSS were specific to the cell types studied—the human kidney epithelial lines IHKE and HPCT or the neuroblastoma lines SH-SY5Y and SK-SNSH. By RLM-RACE, two novel TSS were found in the *KIBRA* 5′ flanking regions of both IHKE and SH-SY5Y cells, 415bp and 153bp upstream of the already annotated TSS (designated TSS1c and TSS1b respectively) [82]. The authors then identified two additional potential TSS in the first intron of *KIBRA* in the IHKE and HPCT cell lines as well as kidney biopsies, but not in the neuroblastoma cell lines or neuroblastoma biopsies. These sites were designated TSS2 and TSS3 and were subsequently found to generate two novel alternative exons with a length of 328bp and 205bp that were termed exons 2a and 2b, respectively [82]. Guske et al. also discovered that *KIBRA* expression is regulated by the constitutively active core promoter P1 and three alternative promoters (P1b, P2, and P3) in kidney cells [82]. Specifically, they detected two distinct promoter regions that directed significant transcriptional activity for TSS1b and TSS1c (P1a and P1b) and found that the intronic promoter regions P2 and P3 drove TSS2 and TSS3. Finally, the study found that the transcription factor TCF7L2 was involved in regulating *KIBRA* expression. Previous ChIP-on chip analysis has shown *YAP1* and *TEAD1* to be targets of TCF7L2 [83] and Guske et al. showed that the P1a and P2 promoter regions they identified contained binding sequences for TCF7L2. Subsequently they observed enhanced transcriptional activity for *KIBRA* promoter deletion constructs when co-transfected with full length TCF7L2, *YAP1* and *TEAD1* in kidney cells. Interestingly, they also observed synergistic transcriptional activation effects when co-transfecting *KIBRA* promoter deletion constructs with TCF7L2, *YAP1* and *TEAD1*. Taken together, these data provide evidence that *KIBRA* expression is driven by an alternative promoter system, which is differentially activated by the transcription factor complex of TCF7L2, YAP1, and TEAD1 and may hint at a possible feedback regulatory mechanism between downstream and upstream components of the Hippo pathway. 

It is also possible that external and environmental factors may be linked to AS that can affect the Hippo pathway. A recent paper from the Zhang group [84] shows that the toxic metal cadmium, which can enter the body through contaminated water, air and food, and can readily accumulate in the body and particularly the ovaries, can affect kit ligand (*KITL*) pre-mRNA AS of exon 6 changing the ratio of kitl1/kitl2 mRNA. Treatment of murine ovarian cells with cadmium was shown to affect the expression of 29 microRNAs (miRNAs) associated with the *KITL* gene [84]. Gene ontology analysis showed that the target genes of these 29 miRNAs were enriched in biological processes such as cellular metabolism, signal transduction, cell cycle and proliferation, differentiation and migration. Interestingly, Kyoto Encyclopedia of Genes and Genomes (KEGG) pathway analysis showed that some of the target genes of these 29 miRNAs were enriched in the Hippo pathway, providing new evidence that environmental factors can affect the Hippo pathway by modulating AS [84]. 

## 5. The Hippo Pathway and Disease

Given the range of inputs that can regulate Hippo pathway activity and the vast array of biological processes that are associated with Hippo signalling, tight control of this pathway is essential for normal functioning of tissues and homeostatic processes. To highlight the importance of controlling Hippo signalling, we will briefly outline some examples of disease states where aberrant Hippo signalling has been found to play a role. Altered YAP/TAZ levels have been implicated in a variety of cancers including breast, pancreatic, prostate, liver, lung, bladder, gliomas, melanomas, sarcomas, colorectal, ovarian, gastric and head and neck cancers (reviewed in [1]). In some instances of cancer, it has been shown that inactivity of upstream Hippo pathway members (e.g., through mutations in *NF2/MERLIN*, *LATS1*, *LATS2* or *SAV1*) can lead to over-activity of YAP/TAZ, which can lead to tumour initiation [85,86,87,88].

However, mutations in core upstream regulators of the Hippo pathway in cancers where YAP/TAZ activity is high are surprisingly infrequent, suggesting other mechanisms leading to the stabilisation of YAP/TAZ may be more important in these contexts [1]. Such mechanisms could include promoter hypermethylation, mutation and amplification [89]. YAP/TAZ are able to drive many of the traits associated with cancer cells including irregular cell proliferation [61], increased cell survival [62,90,91], expansion of the cancer stem cell population driving the tumour [92,93,94,95], and chemoresistance and metastasis by regulating the tumour microenvironment [96]. Interestingly, in pancreatic cancer it has been shown that YAP/TAZ can compensate for activating mutations in the classical oncogenic driver, *KRAS*, suggesting a means for the cancer to bypass oncogenic MAPK signalling and the associated therapeutic approaches [97], highlighting the need for therapies targeted at YAP/TAZ for cancer.

Altered Hippo pathway signalling has also been linked to cardiovascular diseases [98,99], neurodegenerative disorders (e.g., [100]) and ocular diseases. A causal mechanism for arrhythmogenic right ventricular cardiomyopathy (ARVC) was recently shown to be reduced transcriptional activity of YAP due to phosphorylation by upstream Hippo components [101]. A pathological hallmark of ARVC is the replacement of cardiomyocytes by fibro-adipocytes, predominantly in the right ventricle. Knockdown of LATS1/2 in ARVC model myocyte lines restored adipogenesis to levels observed in normal myocytes [101]. The Hippo pathway is also implicated in Holt–Oram syndrome, which involves defects of the heart due to mutations in the transcription factor TBX5 [102]. These mutations prevent TBX5 binding YAP/TAZ resulting in congenital heart defects [103]. YAP/TAZ have been reported to regulate gene transcription induced by AβPP, the precursor of amyloid β, which is implicated in driving Alzheimer’s disease [104]. YAP/TAZ regulate this transcription through interactions with MINT1 and MINT3. Furthermore, *MST1* is a key player in amyotrophic lateral sclerosis (ALS). *MST1* activity is increased in mouse models of ALS and when *MST1* is knocked out in these mice they display increased motor neuron viability with delayed symptom onset accompanied by extended survival [105]. Increased expression of *MOB1* and *LATS1* is observed in early retinal degeneration, which is associated with alterations in photoreceptor proliferation and deregulation of cell cycle genes [106]. Keratoconus, in which the cornea progressively thins and becomes cone-shaped, has been linked to changes in collagen synthesis and maturation [107] associated with downregulation of core Hippo pathway members. Ocular coloboma, the most common eye birth defect, is characterised by an optic fissure closure defect [108]. Williamson et al. found through exosome sequencing of affected individuals the presence of two different heterozygous nonsense mutations in *YAP1* (c.370C > T (p. Arg124*) and c. 1066G > T (p. Glu356*)) [108]. The study found an alternative TSS in intron 1 of *YAP1* and concluded that c.370C > mutations occurring in these alternative *YAP1* transcripts resulted in no NMD, suggesting the process of optic fissure may be sensitive to YAP1 dosage [108].

Polycystic kidney disease (PKD) is a serious condition where cysts form throughout the kidneys. Frequently this is associated with inactivating mutations in *PKD1* and *PKD2* [109]. The YAP1 target gene, *FJX1*, which is required for tubular regeneration in the kidneys following injury, is unchanged in expression in *PKD1* knockout mice [110]. However, unlike in normal kidneys, YAP has been found to persist in the nucleus following recovery leading to cyst formation [110]. Somewhat surprisingly, TAZ plays a different role in PKD progression. TAZ complexes with PC2, the product of *PKD1*, leading to ubiquitination and degradation of PC2 [109]. Consequently, loss of TAZ leads to accumulation of PC2 and development of PKD [109,111,112].

Clearly then from the examples above, there is merit in targeting the Hippo pathway therapeutically for disease amelioration. In the future it will be interesting to elucidate whether specific AS variants of Hippo pathway members may play a role in disease (Table 2), and it is likely that as research in this area progresses, any Hippo pathway AS variants found to be responsible for disease states will provide new therapeutic targets. Several AS variants of YAP, namely *YAP1*β, *YAP1*γ and *YAP1*δ have disruptions to the amino acid sequence of the leucine zipper region encoded within the TAD domain of YAP, that might render it thermodynamically unstable [69]. Since the formation of leucine zippers is important for the interaction of YAP with other proteins, it is likely that these YAP isoforms will have problems associating with some of the common cellular binding partners of YAP [69]. Therefore, it could be speculated that a shift in splicing towards these β, γ and δ variants would have consequences in terms of signal transduction through YAP within the cell but may also provide a mechanism for allowing tissue-specific differences in YAP signalling. Deregulation of the production of these various YAP isoforms in specific tissues at specific times could have negative outcomes leading to developmental defects or other disease states later in life. Interestingly, in the corresponding region of TAZ it was found that no AS takes place [69], suggesting that evolutionary differences between the paralogues may allow for some compensatory effects between YAP and TAZ should one of these paralogues be affected by defective splicing. 

Several pharmacological inhibitors have been designed and tested in an effort to manipulate the activity of the Hippo pathway. Verteporfin is a small molecule inhibitor that directly binds YAP to block its interaction with TEAD transcription factors and thusly its transcriptional activity [113,114]. In mice livers overexpressing YAP or with inactivation of *NF2/MERLIN*, verteporfin was able to suppress the liver overgrowth observed in non-treated mice and the treated livers presented with reduced cell proliferation [113]. Dobutamine, which is a G-protein-coupled β-adrenergic receptor agonist, has been shown to cause dose-dependent cytoplasmic accumulation of GFP-tagged YAP through phosphorylation at the main serine-127 phosphorylation site of YAP, and hence transcriptional inactivity [115]. In the case of ivermectin, an antiparasitic drug, which is able to decrease YAP nuclear accumulation and its transcriptional activity in vitro, the mechanism of action remains unknown, but this compound also has significant effects on tumour growth in vivo [116]. A host of compounds targeting cellular cytoskeletal components (e.g., F-actin, Rho, ROCK and non-muscle myosin), and thus the mechanotransduction pathway that feeds into Hippo signalling have also been shown to have potentially therapeutically exploitable effects on YAP/TAZ activity. This is pertinent given the emerging importance of mechanotransduction in the progression of numerous diseases [117]. For example, latrunculin B and cytochalasin D (both targeting F-actin) have been shown to reduce YAP nuclear localisation via enhanced activity of LATS [91,118,119]. Y27632 (ROCK inhibitor), Blebbistatin (non-muscle myosin inhibitor) and botulinum toxin C3 (Rho inhibitor) all block nuclear localisation of YAP/TAZ with a concomitant reduction in target gene transcription [43,91,118], with botulinum toxin C3 also having been shown to increase phosphorylation of YAP/TAZ and reduce LPA- and S1P-induced YAP/TAZ dephosphorylation [91].

Undoubtedly the systemic delivery of some of these compounds could have significant off-target effects given the ubiquitous presence of some of their targets, as well as the widespread nature of YAP/TAZ and Hippo signalling within the body. Precisely targeted delivery would be required to ensure such inhibitors did not disrupt normal homeostatic approaches in surrounding healthy normal tissues. The requirements for a considered approach to therapeutically targeting the Hippo pathway are supported by examples showing that YAP deletion in the mouse intestine can cause WNT hypersensitivity leading to enhanced injury-induced stem cell expansion and hyperplasia [120]. As discussed, GPCRs are implicated in Hippo signalling, with Gα12/13, Gαq/11 and to a lesser extent Gαi/o being demonstrated as potent activators of YAP/TAZ [46], whereas Gαs-coupled GPCR agonists increase LATS1/2 activity leading to inhibition of YAP/TAZ activity [121]. Interestingly, Gαs-coupled GPCR signalling pathways can also play a pro-tumorigenic role in some tumour types [122]. Therefore, compounds targeted against GPCR pathways for therapeutic means could actually increase YAP/TAZ activity with potentially harmful outcomes.

Therefore, inhibitors/agonists that alter only the disease state-specific aspect of Hippo signalling or YAP/TAZ activity may be preferable. In particular, therapies that target the most downstream aspect of Hippo signalling, i.e., YAP-TEAD activity could hold significant therapeutic promise [113]. One such targeted approach may be to direct therapies at disease-specific or sub-optimal splice variants of the Hippo pathway, such as the full length *TEAD4* isoform seen in lung and colon cancer [65] and the β, γ, and δ isoforms of YAP with affected TAD domains [69]. Given that the majority of the core components of the Hippo pathway have splice variants (Figure 2), it seems plausible that a deregulation in spatio-temporal splicing of these core members could lead to the production of splice variants with deleterious effects. Such harmful splice isoforms would provide the opportunity to target disease-causing splice variants without affecting the functioning of normal Hippo signalling, thus minimising any potential side effects. We will now discuss the prospect of targeting changes in splicing in the Hippo pathway as a means of treatment for disease.

## 6. Approaches to Target Alternative Splicing Therapeutically in the Hippo Pathway

Evidence is now emerging for disrupted AS in the Hippo pathway contributing to disease. A study by Bueno et al. performed a comprehensive genomic analysis of mutations found in malignant pleural mesothelioma (MPM) tumors [87]. Exome analysis found *NF2/MERLIN* to be significantly mutated in MPMs and interestingly *NF2/MERLIN* was found to be frequently inactivated by recurrent gene fusions (e.g., with *GSTT1*) and splicing alterations, namely in-frame deletions predicted to produce a non-functioning protein [87]. The authors found in-frame deletions of the FERM-central, FERM-PH-like and FERM-C domains together or the FERM-PH-like domain alone [87]. Such studies highlight the pertinence of therapeutically targeting disease-specific Hippo pathway splice variants. In support of such a strategy, the use of cancer-specific splice variants for a variety of genes, as biomarkers and therapeutic targets is already becoming recognised for various cancers [123]. Approaches could make use of techniques to bias splicing towards producing normal or disease suppressing variants of the proteins in question. For example, splicing could be modulated in cases where the inclusion of a cassette exon is associated with negative outcomes or where aberrant splicing leads to a protein isoform with a dominant-negative function over the *normal* variant. Or indeed in cases where the gene/protein causing disease is overexpressed, such therapeutic approaches could be used to negate levels of gene expression by introducing premature termination codons (PTCs) into the mRNA produced and thus NMD.

**Figure 3:**
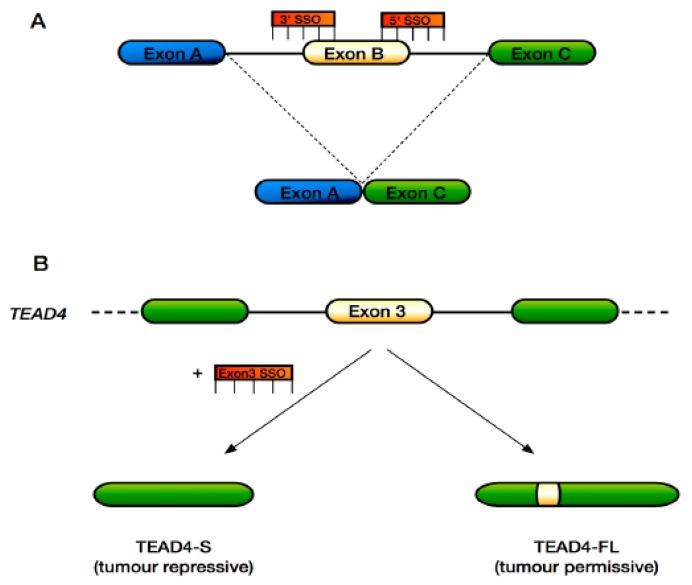
Mechanism of action of splice-switching oligonucleotides. (**A**) By targeting the 3′ acceptor or 5′ donor splice sites, targeted exons can be excluded from the mRNA product; (**B**) A potential approach to target exon 3 of *TEAD4* to produce TEAD4-S, a tumour-suppressive TEAD4 isoform in the context of lung and colon cancer. The principles of this approach could be applied to other diseases to target genes with splicing defects.

Splicing-switching oligonucleotides (SSOs) are a class of antisense oligonucleotides that work at the pre-mRNA level to modify splicing [124] (Figure 3A). Therefore, SSOs can be used to modify the expression of disease-related genes and their protein products. SSOs are in clinical trials for targeting neuromuscular disease genes including *SMN2* [125,126,127,128,129] validating their use as an approach to treat previously intractable diseases such as SMA and DMD. Furthermore, SSOs have been used to inactivate various cancer-associated genes such as *HER2*, *HER4*, *FLT-1*, *MDM4*, *STAT3* or *MCL-1* [124,130,131,132,133,134,135]. 

In the context of the Hippo pathway, SSOs could be used to affect splicing of the *TEAD4* gene in relevant cancers, causing skipping of exon 3 to produce the truncated tumour suppressor variant TEAD4-S (Figure 3B), which has been shown to suppress tumour growth in vivo and has been associated with improved survival in lung and colon cancer patients [65]. Indeed, it might also be possible to correct the aberrant splicing of *NF2/MERLIN* reported in MPM tumours [87] that results in in-frame deletions and a non-functioning protein. Here, SSOs could be targeted to cause retention of the FERM-central, FERM-PH-like and FERM-C domains that are deleted. In the case of breast cancer, where certain ERBB4 isoforms activate YAP target genes to promote cell proliferation and invasion, SSOs may be used to steer splicing towards the production of ERBB4 isoforms that do not result in YAP transcriptional output. Finally, the functions of the various YAP splice variants are still being elucidated. *YAP1*γ has been shown to promote proliferation, colony formation and EMT as well as protect against apoptosis in MCF10A cells [54,136,137]. *YAP1*γ in vivo has been shown to cause liver overgrowth [62]. Conversely, over-expression of the *YAP1*α variant in the UMSCC-11A squamous cell carcinoma cell line caused increased cell death [138]. Consequently, targeting YAP isoform production with SSOs could also have clinical gains. As additional Hippo pathway disease-specific splice variants are identified, the list of potential therapeutic SSO targets should grow.

As discussed above, there are several inhibitors targeted against the Hippo pathway, mostly against YAP. To the best of our knowledge, there are currently no reports of inhibitors targeted against specific splice variants of the YAP protein or other Hippo pathway member protein isoforms. As more Hippo pathway splice variants are identified, their functions elucidated and associated with disease, this is likely to change. As the splicing factors and kinases regulating splicing in the Hippo pathway become more established, it may also be possible to target these pharmacologically, as has been done for CDC Like Kinase 1 (CLK1) with TG693 in the context of DMD [139]. There is some evidence to suggest that serine/arginine-rich splicing factor 1 (SRSF1) and YAP can interact [140]. The metastasis-associated lung adenocarcinoma transcript 1 (*Malat1*) is over-expressed in several disease states and YAP was found to upregulate *Malat1* in liver cancer, whereas SRSF1 was found to have an opposing effect [140]. SRSF1 was found to inhibit YAP activity by blocking its binding with TCF/β-catenin on the *Malat1* promoter [140]. YAP over-expression could inhibit nuclear localisation of both SRSF1 and YAP due to their binding with angiomotin, mitigating the inhibitory role of SRSF1 on *Malat1* expression resulting in enhanced transwell migration of hepatocarcinoma cell lines as well as enhanced tumour growth of hepatocarcinoma cell lines in xenograft models [140]. Whether this interaction between SRSF1 and YAP remains under normal physiological conditions remains to be seen, but the authors speculate that disrupting the interaction between SRSF1 and YAP may be a viable therapeutic target to modulate the Hippo pathway in the context of liver cancer [140].

## 7. Conclusions

AS is a very important regulator of multicellular eukaryotic gene function, the deregulation of which contributes to many disease states, including cancer. Likewise, the Hippo pathway, a master regulator of organ size and the associated biological processes is also highly implicated in disease. Given the evolutionary and biological significance of this signalling pathway, it is unsurprising that AS is emerging as a means to offer both an extra layer of regulation of this pathway as well as a means for the pathway to have diverse functions in different tissues. As our knowledge of the role of AS in the Hippo pathway expands, we should be able to identify valuable new therapeutic targets specific to disease states.

## Figures and Tables

**Table 1:** Summary of splicing events in the Hippo pathway. Hippo pathway members shown in bold belong to the core cassette of the Hippo pathway.

Hippo Pathway Component	Number of Splice Variants	Potential Consequences of Splice Variant for Hippo Pathway Signalling	Refs.
*FAT4*	3 (2 protein coding)	Not yet known	
*CD44*	39 (27 protein coding)	Not yet known	
*FRMD6*	18 (10 protein coding)	Not yet known	
*KIBRA*	16 (5 protein coding)	Not yet known	
*NF2*/*MER*	11 (10 protein coding)	Not yet known	
*TAO*	6 (3 protein coding)	Not yet known	
***MST1*/*2***	21 (2 protein coding)	C-terminal truncated version of MST1 consists only of kinase domain, full-length MST1 isoform has C-terminal regulatory region and homodimerisation SARAH domain. Truncated isoforms of MST1 may not homodimerise with MST2.	[66]
***SAV1***	6 (5 protein coding)	Forms a complex through its C-terminal SARAH domain with MST1 and MST2. Truncated isoforms may not complex.	[67]
***RASSF***	10 (5 protein coding)	Forms a complex through its C-terminal SARAH domain with MST1 and MST2. Truncated isoforms may not complex.	[67]
***NDR1*/*2***	2 (2 protein coding)	Not yet known	
*MAP4K*	15 (5 protein coding)	Not yet known	
*MOB1*	6 (1 protein coding)	Asp63 and Lys104/Lys105 of MOB1 are key residues for binding with LATS1/2. Splice isoforms lacking these residues may not bind LATS1/2.	[68]
***LATS1***	7 (4 protein coding)	AS variants could negatively affect interactions with NF2 or MOB1, damage the catalytic domain of LATS1 or interfere with activating phosphorylations from upstream kinases.	[68]
***LATS2***	2 (1 protein coding)	AS variants could negatively affect interactions with NF2 or MOB1, damage the catalytic domain of LATS2 or interfere with activating phosphorylations from upstream kinases.	[68]
***YAP***	11 (9 protein coding)	β, γ and δ isoforms have altered leucine zippers within TAD domain, potentially affecting protein interactions. YAP∆C isoforms lack PDZ-binding motif so may not translocate to nucleus. Both changes may reduce YAP transcriptional activity.	[69,70,71,72,77]
***TAZ***	24 (8 protein coding)	Not yet known	
*ZO-1/2*	12 (8 protein coding)	Not yet known	
*α-cat*	44 (27 protein coding)	Not yet known	
*β-cat*	15 (10 protein coding)	Not yet known	
*PTPN14*	5 (2 protein coding)	Not yet known	
*SCRIB*	8 (5 protein coding)	Not yet known	

MST1: mammalian Ste20-like kinase-1; MOB1: kinase co-activator; LATS1/2: large tumour suppressor kinase 1/2; AS: alternative splicing; NF2: neurofibromin-2; TAD: transcriptional activation domain; YAPΔC: neuronal-specific YAP isoforms (ins13, ins25, and ins61).

**Table 2:** Diseases and phenotypes caused by changes in splicing in Hippo pathway components.

Hippo Pathway Component with Changes in Splicing	Associated Disease/Phenotype	References
*NF2/MERLIN* (in-frame deletions predicted to produce a non-functioning protein)	Malignant pleural mesothelioma	[87]
*YAP* (use of alternative TSS in intron 1 of *YAP1* accompanied by c.370C > mutation)	Ocular coloboma	[108]
*YAP* (*YAP1*γ—disruptions to the amino acid sequence of the leucine zipper region encoded within the TAD domain)	Promotes proliferation, colony formation and EMT as well as protecting against apoptosis in MCF10A cells. Causes liver overgrowth in vivo.	[54,62,136,137]
*YAP* (*YAP1*α—shortest YAP1 isoform)	Causes increased cell death in UMSCC-11A squamous cell carcinoma cell line	[138]
*TEAD4* (TEAD4-S—truncated version lacking the N-terminal DNA-binding domain)	Tumour suppressive in the context of lung and colon cancer	[65]

EMT: epithelial-to-mesenchymal-transition; TSS: transcription start site.
